# Virtual Non-Contrast Reconstructions Derived from Dual-Energy CTA Scans in Peripheral Arterial Disease: Comparison with True Non-Contrast Images and Impact on Radiation Dose

**DOI:** 10.3390/jcm14155571

**Published:** 2025-08-07

**Authors:** Fanni Éva Szablics, Ákos Bérczi, Judit Csőre, Sarolta Borzsák, András Szentiványi, Máté Kiss, Georgina Juhász, Dóra Papp, Ferenc Imre Suhai, Csaba Csobay-Novák

**Affiliations:** 1 Department of Interventional Radiology, Heart and Vascular Center, Semmelweis University, Városmajor utca 68, 1122 Budapest, Hungarymate.kiss@siemens-healthineers.com (M.K.); csobay.csaba@semmelweis.hu (C.C.-N.); 2 Siemens Healthcare, 1143 Budapest, Hungary

**Keywords:** peripheral arterial disease, computed tomography angiography, dual-energy computed tomography angiography, dual-energy, virtual non-contrast image reconstruction

## Abstract

**Background/Objectives**: Virtual non-contrast (VNC) images derived from dual-energy CTA (DE-CTA) could potentially replace true non-contrast (TNC) scans while reducing radiation exposure. This study evaluated the image quality of VNC compared to TNC for assessing native arteries and bypass grafts in patients with peripheral arterial disease (PAD). **Methods**: We retrospectively analyzed 175 patients (111 men, 64 women, mean age: 69.3 ± 9.5 years) with PAD who underwent lower extremity DE-CTA. Mean attenuation and image noise values of TNC and VNC images were measured in native arteries and bypass grafts at six arterial levels, from the aorta to the popliteal arteries, using circular regions of interest (ROI). Signal-to-noise ratios (SNRs) and contrast-to-noise ratios (CNRs) were calculated. Three independent radiologists evaluated the subjective image quality of VNC images compared to baseline TNC scans for overall quality (4-point Likert scale), and for residual contrast medium (CM), calcium subtractions, and bypass graft visualization (3-point Likert scales). Radiation dose parameters (DLP, CTDIvol) were recorded to estimate effective dose values (ED) and the potential radiation dose reduction. Differences between TNC and VNC measurements and radiation dose parameters were compared using a paired *t*-test. Interobserver agreement was assessed with Gwet’s AC2. **Results**: VNC attenuation and noise values were significantly lower across all native arterial levels (*p* < 0.05, mean difference: 4.7 HU–10.8 HU) and generally lower at all bypass regions (mean difference: 2.2 HU–13.8 HU). Mean image quality scores were 3.03 (overall quality), 2.99 (residual contrast), 2.04 (subtracted calcifications), and 3.0 (graft visualization). Inter-reader agreement was excellent for each assessment (AC2 ≥ 0.81). The estimated radiation dose reduction was 36.8% (*p* < 0.0001). **Conclusions**: VNC reconstructions demonstrated comparable image quality to TNC in a PAD assessment and offer substantial radiation dose reduction, supporting their potential as a promising alternative in clinical practice. Further prospective studies and optimization of reconstruction algorithms remain essential to confirm diagnostic accuracy and address remaining technical limitations.

## 1. Introduction

Peripheral arterial disease (PAD) is a complex vascular disorder characterized by steno-occlusive lesions of the peripheral arteries, primarily developing through atherosclerosis. The condition most commonly affects the lower extremity arteries from the aortic bifurcation to the pedal arteries [1]. Atherosclerotic PAD is a manifestation of systemic atherosclerosis, often presenting with additional atherosclerotic comorbidities, and it is reported to be the third leading cause of atherosclerotic morbidity after coronary artery disease and cerebrovascular disease [2,3,4,5]. The global prevalence of PAD has been on the rise, affecting more than 200 million people worldwide [6]. Thus, atherosclerotic PAD in the lower extremity has become a major contributor to morbidity and mortality, with a significant increase in the global burden of the condition [2].

PAD has a broad spectrum of clinical manifestations ranging from asymptomatic disease, atypical leg pain, to intermittent claudication, and chronic limb-threatening ischemia [7]. As a chronic and slowly progressive disease, PAD is often masked by collateral circulation development and remains clinically silent for extended periods, leading to delayed diagnosis and therapeutic interventions [8,9]. Thus, early and accurate diagnosis is crucial to improving patient outcomes. While traditional diagnostic methods, such as clinical evaluation, physical examinations, along with the ankle–brachial index (ABI) test, remain the initial step in current clinical practice, and various imaging techniques, including ultrasound (US), computed tomography angiography (CTA), magnetic resonance angiography (MRA) and digital subtraction angiography (DSA), are essential in the diagnostic evaluation of the obstructive disease [4]. Current ESC and AHA/ACC guidelines recommend a comprehensive, indication-based imaging approach tailored to specific clinical settings, such as screening, anatomical mapping, treatment planning, interventional guidance (with DSA), and long-term follow-up [10,11]. Moreover, selecting a particular imaging technique depends on its availability, patient comorbidities, cost-effectiveness, and physician preference [4].

CTA is a widely used, non-invasive imaging modality for evaluating PAD, typically using biphasic protocols with unenhanced and contrast-enhanced early arterial phases [10,11]. CTA is easily accessible and offers rapid image acquisition with high spatial resolution. The option of multi-planar reconstructions of arteries or grafts with CTA enables precise characterization of lesion length, location, and vascular or graft morphology. These capabilities are essential for accurate treatment planning and postprocedural follow-up [12,13,14,15,16]. Nevertheless, the risks associated with radiation exposure have remained concerning [17]. CTA employs significant radiation doses, contributing to patients’ cumulative exposure to ionizing radiation [18,19]. The rapid increase in CT scan usage over recent decades—despite their generally favourable benefit-to-risk ratio—has raised concerns about cumulative radiation exposure, highlighting the importance of dose reduction strategies [19]. In patients with PAD, who often present with multiple comorbidities and undergo numerous ionizing radiation-based diagnostic procedures, minimizing radiation exposure—particularly from lower extremity CTA scans—is essential for enhancing long-term patient safety and adhering to radiation protection principles such as ALARA (As Low As Reasonably Achievable) [20].

With the development of third-generation CT scanners, dual-energy CT (DE-CT) has become a novel technique in CT imaging [21,22]. DE-CT can simultaneously capture two sets of data at different X-ray tube potentials and takes advantage of the unique attenuation values at different X-ray energy levels [4]. This leads to a process called material decomposition, allowing more precise quantification of materials. By applying post-processing algorithms, an iodine map can be produced and subtracted from contrast-enhanced images, hence the appearance of non-contrast scans can be simulated without the need for true non-contrast (TNC) acquisitions, creating “virtual non-contrast” (VNC) images [23,24].

Reconstructing VNC images with DE-CT presents a novel opportunity to improve patient safety by reducing radiation exposure. VNC reconstructions have the potential to significantly reduce the radiation dose if image quality and diagnostic accuracy comparable to TNC acquisitions are confirmed. Several studies have investigated the application of VNC reconstructions [25,26,27,28], yet there is a lack of data regarding their performance in PAD assessment. VNC images have been shown, in a relatively small patient population, to be potentially suitable for PAD detection [29]. Additionally, no article has been published studying the accuracy of VNC images in evaluating lower extremity bypass grafts.

This study aims to provide an assessment of VNC image quality derived from DE-CT early arterial scans (DE-CTA), in contrast to TNC images, for the diagnostic evaluation of PAD in native arteries and bypass grafts, and to evaluate the potential impact of replacing TNC scans with VNC reconstructions on radiation dose.

## 2. Materials and Methods

The study was designed as single-center retrospective research and was conducted in accordance with the Declaration of Helsinki and with the approval of the Semmelweis University Regional and Institutional Committee of Science and Research Ethics on 31 January 2023 (SE RKEB number: 173/2023).

### 2.1. Case Selection

**Figure 1:**
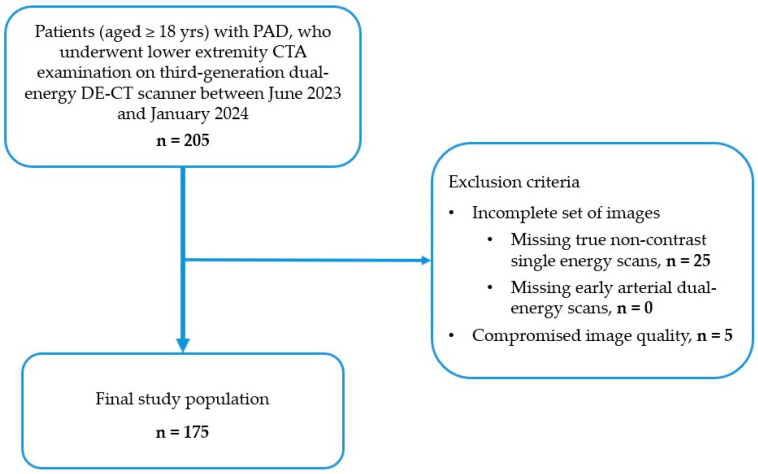
Flowchart illustrating patient selection, showing patient inclusion and exclusion criteria for this study. Inclusion criteria comprised adult (≥18 yrs) patients with PAD who underwent lower extremity DE-CTA examination at our institution between June 2023 and January 2024. Exclusion criteria included incomplete set of images (missing true non-contrast or arterial phases), and compromised image quality (e.g., motion or metallic artifacts, bad contrast timing). Of the initially included 205 patients, 30 patients were excluded. Eventually, 175 patients were enrolled in this study. PAD, peripheral arterial disease; CTA, CT angiography; DECT, dual energy CT.

Between June 2023 and January 2024, patients with PAD who were scheduled to undergo vascular evaluation by lower-extremity DE-CTA for suspected limb ischemia or pre-operative treatment planning in the Heart and Vascular Center of Semmelweis University were enrolled in this study by the following inclusion criteria (see Flowchart in Figure 1):

- Age ≥ 18 years.
- Contrast-enhanced lower extremity CT examination comprising unenhanced and dual-energy early arterial phases.

The exclusion criteria were the following:

- An incomplete set of images, including true non-contrast and early arterial dual-energy CT scans.
- Compromised image quality, due to the presence of motion or metallic artifacts, as well as bad contrast timing.

Every scan was clinically indicated; no scan was performed for the purpose of this study only. Case selection is shown in Figure 1. Demographic and clinical data of the study population were collected.

### 2.2. CT Scan Protocol

All scans were conducted using a third-generation DE-CT scanner (Siemens Somatom Force, Siemens Healthineers, Erlangen, Germany). The patients were positioned supine and feet first for a craniocaudal scan from the abdominal aorta to the feet, while holding their breath after a deep inhalation. The intravenous contrast medium (CM) was injected through an 18-gauge cannula and placed in a forearm or antecubital superficial vein.

After completing the non-contrast CT scan, early arterial DE-CTA was acquired. The bolus tracking acquisition was performed with 120 kVp (CARE Bolus, Siemens Healthineers, Erlangen, Germany). The trigger threshold was set for 250 Hounsfield units and a 7 s delay. A total of 1 mL/kg of contrast material (Iomeron 400), followed by 45 mL of saline, was injected at a standardized flow rate between 3.5 and 4.5 mL/s. The exact rate was typically set at 4 mL/second unless limited by intravenous access, in which case it was adjusted based on our clinical experience. A tube current was adapted by automatic exposure control (CareDose 4DTM, Siemens Healthineers, Erlangen, Germany) depending on patient characteristics.

The scanning parameters, both for non-contrast and DE-CTA acquisition, were chosen in relation to our clinical and diagnostic experience. The CT scan parameters are summarized in Table 1.

### 2.3. DE-CTA Image Reconstruction

A medium sharp convolution kernel (Qr36) was used for the reconstruction of a transverse low- and high-kVp DE-CTA dataset. Image post-processing was performed by a patented algorithm on a specific DE-CT workstation (Syngo MMWP version VA60; Siemens Healthineers, Erlangen, Germany), generating virtual non-contrast images from DE-CTA scans. Specifically, VNC images were reconstructed using the syngo.via CT Dual Energy workflow (Siemens Healthineers, Erlangen, Germany), applying the syngo.via CT DE Virtual Unenhanced application. All reconstructions were performed with default vendor-recommended decomposition parameters. Following the quantification of the iodine content of each voxel and the generation of an iodine map with the algorithm, VNC images were reconstructed with 1 mm slice thickness.

### 2.4. Image Quality Assessment

Image analysis was performed on the dedicated picture archiving and communication system (PACS) workstation (Syngo.via, Siemens Healthineers, Erlangen, Germany). DE-CTA, VNC, and TNC images were reviewed for the image quality analysis.

#### 2.4.1. Quantitative Analysis

**Figure 2:**
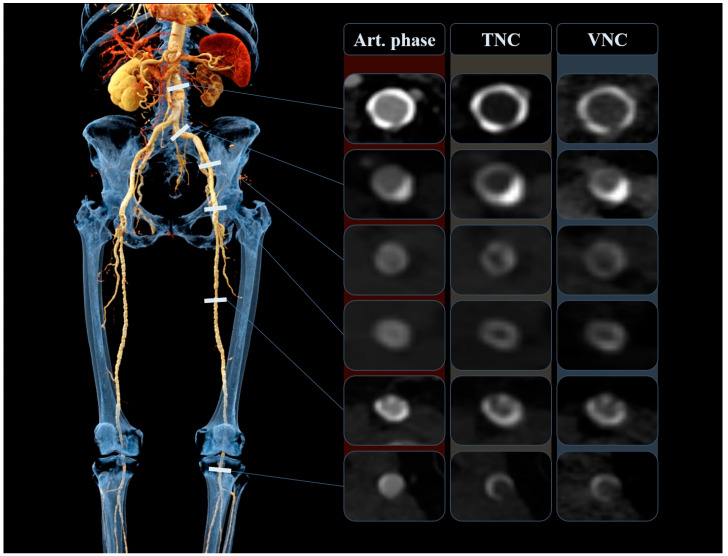
Three-dimensional cinematic volume rendered reconstruction (**left**) and axial image slices (**right**) from a representative patient, undergoing lower extremity DE-CTA. Axial slices from the arterial phase (art. phase), true non-contrast (TNC), and derived virtual non-contrast (VNC) images are shown. Quantitative measurements and qualitative analysis were conducted at six arterial levels (from top to bottom): infrarenal aorta, common iliac arteries, external iliac arteries, common femoral arteries, superficial femoral arteries, and popliteal arteries. Art. phase, arterial phase; TNC, true non-contrast; VNC, virtual non-contrast.

The objective measurements were completed by one radiologist (F.É.S.) with 2 years of experience in vascular and interventional radiology. The quantitative assessment included the comparison of attenuation (HU, Hounsfield unit) and image noise between the VNC and TNC images, by placing circular regions of interest (ROI) on both datasets at the exact same anatomical position. Measurements were obtained intraluminally at six arterial segments, namely at the infrarenal aorta, common iliac arteries, external iliac arteries, common femoral arteries, superficial femoral arteries, and popliteal arteries, as shown in Figure 2. Lower extremity bypass grafts were assessed similarly and at the same arterial levels. Mean attenuation and the corresponding standard deviation (image noise) were recorded. The size of the ROI was matched to the size of the vessel lumen. ROIs were made as large as possible, with the aim of avoiding vascular walls, calcifications, plaques, and any other influencing vascular lesions. Occluded, heavily calcified vessels and stented segments were not measured. Additionally, attenuation and noise were assessed in muscle and adipose tissue by placing ROIs in the gluteus medius muscle and the subcutaneous fat tissue around the gluteal region. Subsequently, we calculated the signal-to-noise ratio (SNR) and contrast-to-noise ratio (CNR) with the following two formulas, used in previous studies [30]:

SNR = HU_vessel_/noise

CNR = (HU_vessel_ − HU_muscle_)/noise

noise = SD of vascular attenuation

#### 2.4.2. Qualitative Analysis

The subjective evaluation was performed by three independent radiologists with 15 (C.C.-N.), 11 (F.I.S.), and 6 (Á.B.) years of experience in vascular and interventional radiology. The scoring was performed on a secure institutional digital platform developed for clinical research and biobanking purposes, where structured scoring forms were available for all readers. The images were anonymized and were presented in a randomized order. Each reader was at liberty to adjust the window settings and browse through the entire stack of CT series. Readers were blinded to patient demographic and clinical information, prior radiology reports, and quantitative measurements. However, they were not blinded to image reconstruction type (VNC vs. TNC) because the VNC image quality was compared side-by-side to the standard TNC images [31]. VNC images were evaluated for overall image quality, residual CM, calcium subtractions, and the assessability of bypass grafts. Overall image quality of VNC images was scored according to a 4-point Likert scale (4/4 excellent, equal to the quality of TNC, 3/4 good, little worse/almost the same quality as the quality of TNC, 2/4 moderate but sufficient for diagnosis, moderately worse than the quality of TNC, and 1/4 non-diagnostic, very poor quality compared to the quality of TNC) used in previous studies [29,32]. In the evaluation for residual CM, subtracted calcifications and bypass grafts of each examined arterial level, the right and left lower extremity arteries, were analysed separately. Based on previous studies [33,34,35], 3-point Likert scales were used for the evaluation of each of the three aspects (Table 2). The structure of the qualitative image quality assessment is summarized in Table 2.

### 2.5. Dose Assessment and Effective Dose Calculation

To assess the absorbed radiation dose, dose length product (DLP, mGy·cm) and CT dose index volume (CTDIvol, mGy) values were recorded from the automatically generated dose report, for each patient having complete lower extremity CT scans up to the feet. Consequently, effective doses (ED, mGy·cm) were calculated by multiplying each DLP by the mean of the ED/DLP conversion factor (k, tissue weighting factor) for lower extremity CTA examinations (k_male_ = 0.0056, k_female_ = 0.0068) [36].

For the evaluation of potential radiation reduction, the estimated percentage of dose reduction was calculated by comparing the effective dose of the biphasic non-contrast and contrast-enhanced phase protocol (TNC + DE-CTA) to the effective dose values of the single-phase contrast-enhanced protocol (DE-CTA only), according to the following formula:

radiation dose reduction % = ((mean radiation dose of TNC + DE-CTA) − (mean radiation dose of DE-CTA))/(mean radiation dose of TNC + DE-CTA) × 100%

### 2.6. Statistical Analysis

Data analysis was performed using the Stata Statistical Software (StataCorp. 2023. Stata Statistical Software: Release 18. College Station, TX, USA: StataCorp LLC.), while inter-reader agreement was evaluated with R Statistical Software (v4.4.3; R Core Team 2021). Categorical variables were described as percentages or frequencies, while numerical variables were reported as the mean and one standard deviation (SD). The distribution of the continuous variables was tested using the Shapiro–Wilk test. The data from the quantitative analysis and radiation dose measurements followed a normal distribution. Accordingly, we compared the values of TNC and VNC images and the values of radiation doses using the paired samples *t*-test. A *p*-value of < 0.05 was considered statistically significant. Interobserver agreement was assessed with Gwet’s second-order agreement coefficient (Gwet’s AC2) statistics [37,38,39], using the irrCAC package (version 1.0) in R [40]. According to Altman, AC2s greater than 0.81 were defined to be of excellent (‘very good’) agreement, 0.61–0.80 ‘good’, 0.41–0.60 ‘moderate’, and 0.21–0.40 ‘fair’, and values equal to or less than 0.20 suggested ‘poor’ agreement [41].

## 3. Results

### 3.1. Patient Characteristics

A total of 205 patients were enrolled in the study. A total of 30 participants were excluded because of an incomplete set of CT scans (missing true non-contrast phase, *n* = 25) and compromised image quality (*n* = 5) (Figure 1). Eventually, 175 patients were included, 64 were women, and 111 were men, with a mean age of 69.3 ± 9.5 years. The summary of demographic and clinical characteristics of the study population is listed in Table 3.

The most common comorbidities in the patient population were hypertension (92.4%), hyperlipidaemia (82.5%), and smoking (81.3%). Most patients (87.4%) were scheduled to undergo elective lower extremity CTA and had chronic ischemic symptoms corresponding to Fontaine stage IIb. (*n* = 70). Patients with Fontaine stage I. symptoms (*n* = 14) had previously undergone lower extremity bypass surgery and their ischemic symptoms resolved; however, they were referred for follow-up CT imaging due to suspected complications. In total, 30 patients had undergone lower extremity bypass surgery prior to the DE-CTA examination. The characteristics of the bypass conduits are summarized in Table 4.

### 3.2. Image Quality Assessment

#### 3.2.1. Quantitative Analysis

In native arteries, a significant difference was found between the attenuation and image noise values of TNC and VNC images across all arterial levels. VNC images showed lower attenuation and image noise compared to TNC scans. The highest intravascular attenuation differences were measured at the level of the infrarenal aorta (10.8 ± 7.2 HU). In total, differences between TNC and VNC images were 10 HU or less in 64.9%, and 15 HU or less in 85.3% of all native arterial measurements. The mean ROI areas were 0.66 cm^2^ at the infrarenal aorta, 0.2 cm^2^ at the common iliac arteries, 0.11 cm^2^ at the level of the external iliac artery, 0.12 cm^2^ at the common femoral arteries, 7.11 mm^2^ at the superficial femoral arteries, and 7.22 mm^2^ at the level of the popliteal artery. Unlike in the case of intraluminal measurements, adipose tissue showed a significantly higher attenuation on VNC reconstructions, with a mean difference of 36.1 ± 11.6 HU. The difference in muscle attenuation was not significant (*p* = 0.12). Image noise values of VNC reconstructions were significantly lower in muscle and fat. SNR values were significantly lower on VNC images at the level of the infrarenal aorta, common iliac arteries, and in muscle and fat, whereas SNR measurements were significantly higher on VNC images at the level of the common femoral and popliteal arteries. At the level of the external iliac and superficial femoral arteries, no significant difference was found between SNR values. CNR measurements were significantly lower on VNC reconstructions, compared to TNC scans at each arterial segment. The results of the quantitative comparison of native arteries, muscle, and adipose tissue between TNC and VNC images are summarized in Table 5.

In the case of bypass graft measurements, attenuation values were significantly lower on VNC images at the level of the external iliac, common and superficial femoral, and popliteal arteries. Image noise was significantly lower on VNC images at the level of the infrarenal aorta and common femoral arteries. Overall, 79.2% of all bypass graft measurements were 10 HU or less, and 92.5% of all bypass cases were 15 HU or less. No statistically significant difference was observed in SNR values. CNR measurements were significantly lower on VNC reconstructions at the level of the common femoral and popliteal arteries, compared to TNC. The results of the quantitative comparison of bypass grafts between TNC and VNC images are listed in Table 6.

#### 3.2.2. Qualitative Analysis

In the qualitative assessment, VNC images of 48 cases (11 segments each) were compared to the baseline TNC scans by three experienced radiologists. For overall image quality, the mean score was 3.03 according to the 4-point Likert scale. Compared to the TNC scans, the readers altogether rated VNC images as excellent (score 4/4, equal to TNC) in 5 (3.5%) and good (score 3/4, almost equal to TNC) in 139 (96.5%) cases. No VNC image was indicated to be of moderate quality (score 2/4, moderately worse than TNC) or insufficient for the diagnosis (score 1/4, very poor quality compared to TNC). The score ratings of the overall image quality of VNC reconstructions compared to TNC scans are shown in Table 7.

The overall mean score of CM removal was 2.99 based on the corresponding 3-point Likert scale. A complete removal of CM and equivalence to TNC images (score 3/3) was declared in over 99% of the cases in each arterial region. Incomplete removal of CM affecting smaller areas (score 2/3) was reported once by the same reader (reader2) in each segment (11 segments, aorta, and the five arterial regions on both sides). None of the raters reported highly insufficient CM removal (score 1/3).

In the assessment of calcium subtractions, the overall mean rating was 2.04 according to the 3-point Likert scale. For each arterial segment, in over at least 90% of all cases, the readers reported a smaller amount of calcium subtractions on VNC reconstructions (score 2/3). In 10 (6.94%) cases for the infrarenal aorta, 9 (6.25%) and 7 (4.86%) cases for the right and left common iliac arteries, 7-7 (4.86%) cases for the external iliac arteries, 7 (4.86%) and 5 (3.47%) cases for the right and left common femoral arteries, 4 (2.78%) and 5 (3.47%) cases for the superficial femoral arteries, and 4 (2.78%) and 6 (4.17%) cases for the right and left popliteal arteries, the readers indicated complete depiction of calcified lesions (score 3/3) on VNC images. Altogether, larger amounts of calcium removal (score 1/3) from VNC reconstructions were registered in seven (0.44%) cases, with the most cases at the level of the infrarenal aorta (*n* = 2). The summary of score ratings for residual CM and calcium removal is listed in Table 8.

At the arterial segments where previous bypass surgery was performed, readers scored the bypass graft assessability on VNC images compared to TNC scans with a 3-point Likert scale. The overall mean rating was found to be 3.0, meaning that the depiction of bypass grafts was declared to be of equal quality (score 3/3) between VNC and TNC images in each arterial level, by every reader. There were no cases where the visualization of bypass grafts in VNC images was indicated to be of moderately worse (score 2/3) or poor quality (score 1/3).

Since all readers assigned identical scores for graft visualization on VNC images (score 3/3 in all cases), leading to a lack of variance, inter-reader agreement was not calculated for bypass graft scoring. In the case of overall image quality, residual CM, and calcium subtraction assessment, all AC2s were greater than 0.81, meaning that the agreement between readers was excellent (very good). The results of the inter-reader agreement statistics are summarized in Table 9.

### 3.3. Radiation Dose Assessment

We analysed the radiation data of 167 participants, who had a complete set of lower extremity CT scans up to the feet. The comparison of the radiation data between biphasic (TNC + DE-CTA) and single-phase (DE-CTA alone) scans is shown in Table 10.

Overall, the radiation data (DLP, CTDIvol, ED) of biphasic CT scans were significantly (*p* < 0.0001) higher than the radiation data of single-phase CTA examinations. Thus, with a single-phase DE-CTA scan and VNC reconstruction, a corresponding potential mean effective dose reduction of 36.8% would be achievable.

## 4. Discussion

Our retrospective study evaluated 175 lower extremity DE-CT scans to compare the image quality of VNC images reconstructed from early arterial phase contrast-enhanced CT scans and TNC images in the diagnostic assessment of PAD, including the evaluation of native arteries and lower extremity bypass grafts, as well as to estimate the potential radiation dose reduction linked to single-phase DE-CTA examinations by replacing TNC scans with VNC reconstructions. The main findings of the present study demonstrate that while VNC images showed lower attenuation and noise values compared to TNC scans in both native arteries and in vascular bypass grafts, with a mean attenuation difference of 13.8 ± 9.3 HU or less and mean noise difference of 3.8 ± 2.2 HU or less, the subjective image quality of VNC reconstructions were reported to be good to excellent. Additionally, we found that a single-phase DE-CTA scan with VNC reconstruction would have resulted in a significant radiation dose reduction of 36.8% compared to the standard biphasic protocol.

Several studies aimed to account for VNC images as a suitable substitute for TNC scans in various clinical applications [25,26,27]. However, VNC reconstructions are not yet widely accepted in routine clinical practice. One major question is the longitudinal reproducibility of VNC images across different CT platforms and reconstruction algorithms [42]. Variability in attenuation and image noise values between VNC and TNC images, as well as between VNC datasets generated by different vendors (with different protocol settings, including dose modulation techniques), dual-energy acquisition techniques (e.g., dual-source, rapid kVp-switching, dual-layer spectral detectors), or different post-processing algorithms, raises concerns about the consistency and reliability of these reconstructions over time and across institutions. Moreover, one study found the least consistent reproducibility and the highest inter-scan variation in vascular structures such as the aorta, underscoring the need for further validation of VNC reconstructions in vascular settings [43].

In the case of vascular imaging, we found a total of six previous studies exploring VNC reconstructions from DE-CT datasets as an appropriate alternative to TNC scans. Four studies have explored the use of VNC imaging in acute aortic disease and endovascular aortic repair follow-up, with one also including kidney donor evaluation. While most studies were limited to the aorto-iliac axis, one extended the analysis to the femoral arteries [31,32,44,45]. In these studies, the results of intravascular attenuation and image noise measurements were considerably controversial, with studies reporting no significant differences [44,45], higher attenuation and lower noise [31], and significantly higher attenuation and higher noise [32] values on VNC images compared to TNC scans. Although three studies reported acceptable image quality for VNC, Lehti et al. concluded that VNC reconstructions are not suitable alternatives to TNC scans due to statistically significant attenuation and noise differences, as well as insufficient CM removal in the proximal aortic segment and subtraction of stent structures and calcium. Two studies have been published on lower extremity DE-CTA using VNC images [29,30]. One study investigated the assessment of free fibula flap perforators in patients with maxillofacial lesions and reported no statistically significant differences [30]. The other study was linked to the diagnostic assessment of symptomatic PAD [29]. Floridi et al. found that the attenuation of VNC images was significantly lower at all arterial levels from the abdominal aorta to the popliteal artery, except for the external iliac arteries, and concluded comparable reliability and diagnostic accuracy of VNC reconstructions to TNC scans. However, these studies were conducted in relatively small sample sizes. Based on the quantitative assessment in our study with 175 patients, we found that attenuation values measured in native arteries were significantly lower in VNC images compared to TNC scans at all arterial levels and significantly higher in adipose tissue. The attenuation difference in the muscle was not significant. The differences between intraluminal attenuation measurements of vascular bypass grafts were also generally lower on VNC reconstructions; however, significant differences were only found at the level of the external iliac, common femoral, superficial femoral, and popliteal arteries. Image noise values were significantly lower on VNC images compared to TNC scans at each native arterial segment, in muscle and fat, as well as in bypass grafts at the level of the infrarenal aorta and common femoral arteries. The statistically insignificant differences at the remaining arterial regions are most probably related to the considerably small sample sizes in the bypass subgroup. In a report by Sauter et al. studying the reliability of VNC images obtained with DE-CT, they considered differences in attenuation between VNC and TNC images of 10 HU or less negligible and between 10 and 15 HU acceptable. This study resulted in a difference of 10 HU or less in over 80% and of 15 HU or less in 92% of all measurements [25]. Toepker et al. and Ananthakrishnan et al. found similar results with differences of 10 HU or less in over 75.3% and 75.2% and 15 HU or less in over 91.5% and 92.6% of all cases [26,27]. Consistent with these findings, the present study resulted in negligible (≤10 HU) differences between TNC and VNC images in 64.9%, and acceptable (≤15 HU) differences in 85.3% of all native arterial measurements. The attenuation differences in bypass grafts were negligible in 79.2% and acceptable in 92.5% of all cases. In both native arterial and bypass graft measurements, the mean differences in attenuation were negligible at each arterial level, except for the infrarenal aorta, where they remained within acceptable limits. Regardless of statistical significance, this result indicates appropriate iodine subtraction from VNC reconstructions and suggests that VNC images were of comparable image quality to TNC images, which represent an essential prerequisite for their future diagnostic application. Studies also observed that the highest attenuation differences between TNC and VNC images were recorded in the aorta and fat tissue [25,26]. The cause for the high attenuation difference in fat remains unclear [25,30,31]. The higher difference in the aorta is thought to be related to the high iodine concentration in the vessel, especially in the arterial phase, leading to excessive iodine removal during VNC reconstructions and consequently resulting in lower attenuation values. Thus, using CM with lower iodine concentrations may be an important future direction of research to improve these results [25,29]. Beyond its potential technical benefits, reducing iodine concentrations may offer significant clinical advantages, particularly for patients with PAD who often present with multiple comorbidities, including impaired renal function. In this population, minimizing iodinated CM exposure could help mitigate the risk of renal complications, thereby improving the overall safety of lower extremity CTA examinations.

SNR and CNR metrics for the comparison of VNC and TNC images were only included in one of the aforementioned vascular studies [30]. Wang et al. found no significant differences in SNR and CNR measurements of free-fibula flap perforators, from the popliteal to the peroneal arteries, nor in the SNR values of muscle and fat. Our study found heterogeneous results regarding SNR values for different arterial levels. Specifically, SNR values were lower on VNC images at proximal vascular segments (aorto-iliac) and in muscle and fat, and higher at distal vascular levels (femoro-popliteal), in most segments with significant differences, except for the external iliac and superficial femoral arteries and all bypass grafts regions. This is likely due to progressive reduction in attenuation differences and the relatively stable or slightly increasing image noise differences, measured between VNC and TNC from the aorta distally to the popliteal arteries. CNR values were consistently and significantly lower across all native arterial levels and in bypass grafts at the level of the common femoral and popliteal arteries, which can also indicate the excessive iodine removal from arterial vessels during VNC reconstructions, as described above.

In the 4-point Likert scale of the qualitative assessment, all three readers found the overall image quality to be good to excellent, meaning VNC reconstructions have almost the same or equal quality as TNC images. In line with this, the readers indicated complete CM removal and excellent assessability of bypass grafts. Nevertheless, the removal of calcium content from reconstructing VNC images from DE-CTA scans presents an in-built technical limitation of the algorithm, as previously described [31,32,45]. In the present study, a complete depiction of calcifications was only occasionally reported. In most cases (>90%), smaller calcified areas were removed from VNC images. The material decomposition process of the VNC algorithm examines soft tissue, iodine, and fat. Since calcium is not among the analysed materials, partial calcium removal may occur [31,45]. Although readers mostly reported minor calcium removal, which would not have altered the diagnosis in our study, the subtraction of calcified lesions observed on VNC images may, in certain cases, limit the reliability of VNC reconstructions in the diagnostic evaluation of PAD. Even partial calcium removal can lead to underestimation of stenosis severity, particularly in highly calcified vessels, potentially influencing treatment decisions. The VNC algorithms are based on the principle of iodine subtraction, which is defined as the removal of the iodine signal. This process is similar to image denoising, where the preservation of critical image features is paramount. The concept of ‘feature-preserving loss’, as proposed by Dong et al. in the context of AI-based image denoising [46], may provide a theoretical foundation for enhancing current VNC reconstruction methods. This could help mitigate the limitation of inadvertently subtracting diagnostically relevant structures, such as calcified plaques. Future research should focus on integrating such feature-preserving techniques into VNC algorithms to ensure the preservation of vessel boundaries and calcifications during image reconstructions. This could enhance diagnostic reliability, particularly in complex vascular conditions where the accurate depiction of calcifications is of critical importance. Moreover, other limitations of VNC reconstructions should also be considered when contemplating omitting TNC acquisitions. In addition to calcium removal, previous studies have observed subtractions of stent structures, which may result in diagnostic uncertainty and misinterpretation as stent fractures or stent-related complications [31,32]. Patient movement or vascular pulsation can lead to imperfect voxel registration between low- and high-kVp datasets. These subtraction artifacts may result in residual CM, mimicking the presence of non-existent lesions. Residual CM can also arise in slow-flow vessels and may be of clinical relevance if mistaken for intraluminal hematoma or thrombus [32,47]. These potential clinical risks underscore the need for further research to optimize VNC algorithms and to validate their diagnostic reliability before VNC images can safely replace TNC scans in routine clinical practice.

One of the primary benefits of replacing TNC scans with VNC reconstructions is the potential for reduced radiation exposure. Compared to the biphasic protocol, our study found that a single-phase DE-CTA examination with VNC reconstruction can lead to a mean radiation dose reduction of 36.8%. Moreover, in the case of operated PAD patients, lowering the radiation dose is even more important, since they are usually submitted to regular follow-up CT examinations. In these cases, radiation exposure can further be reduced with VNC reconstructions implicated in routine follow-up.

Our study had several limitations. First, our study was a single-center study with retrospective analysis and a relatively heterogeneous patient population. Second, the sample size of the bypass subgroup was comparatively small. Third, although we have measured intraluminally and excluded occluded or heavily calcified vessels from our quantitative analysis to avoid any influencing effects, we did not explicitly record morphological characteristics of vascular lesions, such as the degree of stenosis and the extent of calcification. Therefore, the impact of these factors on VNC image quality, both in native arteries and in bypass grafts, could not be evaluated, limiting the generalizability of our findings. Fourth, during SNR and CNR calculations, we used the SD of intraluminal vascular attenuation rather than the SD of subcutaneous fat. Given the vascular focus of our study, this method was chosen to reflect local, vessel-specific characteristics affecting image quality, particularly across vascular segments of varying calibers, where the use of a uniform fat-based measurement may lead to non-representative results. This approach is further supported by a previous study evaluating VNC images in lower extremity arteries using the same formulas [30]. Fifth, the mixed results regarding insufficient calcium removal may partly be explained by differences in acquisition parameters between contrast and non-contrast phases, such as faster rotation time, higher pitch, and the use of a lower-resolution reconstruction kernel on the contrast-enhanced scan that could have contributed to suboptimal calcium depiction on VNC reconstructions. Sixth, inter-reader agreement was tested with statistics different from those in other similar studies, which may limit the comparison of the data. Since the distribution of our qualitative data was highly homogenous with minimal to no variance, Gwet’s AC2 was chosen instead of the interclass correlation coefficient to obtain more accurate results. Seventh, although we reported DLP, CTDIvol, and ED, which were calculated from DLP and established tissue weighting factors to account for the varying sensitivity of different tissues to radiation, no stratified analyses were conducted based on patient body habitus, such as BMI. Therefore, variability in patient size may have influenced radiation dose results. Additionally, since VNC reconstructions were generated from the contrast-enhanced dataset of the same examination as the TNC scans, they do not represent independent acquisitions. Consequently, this study serves as a technical validation of the quantitative and qualitative similarity between image types, rather than a direct assessment of the clinical diagnostic accuracy of VNC imaging as an independent diagnostic modality.

Although VNC reconstructions derived from DE-CTA scans are not quantitatively identical to TNC scans in terms of attenuation, image noise, SNR, and CNR, their overall similarity—supported by both quantitative thresholds (e.g., ≤10–15 HU difference) and high subjective image quality ratings—suggests they may be suitable alternatives in clinical practice for reducing radiation exposure in patients undergoing lower extremity CT examinations for PAD. The potential of VNC reconstructions to significantly reduce radiation exposure is especially relevant for patients with PAD, who often require repeated imaging to monitor disease progression or post-interventional outcomes. The implementation of VNC images as a substitute for TNC scans offers a promising opportunity to adhere to ALARA principles and improve patient safety and care without compromising image quality. However, further prospective studies focusing on assessing the diagnostic accuracy of VNC images are warranted to confirm whether they could reliably replace TNC scans in clinical practice and decision-making.

## 5. Conclusions

VNC images demonstrated comparable image quality to TNC images in the evaluation of PAD, both in native arteries and bypass grafts. Given its potential to significantly reduce radiation exposure, generating VNC images may serve as a promising alternative to TNC scans in the assessment and follow-up of PAD. Directions of future research should include prospective studies investigating the diagnostic accuracy of VNC images as an independent imaging technique in PAD evaluation, particularly focusing on lesion detection, grading, and treatment decision-making. Further optimization of acquisition and reconstruction parameters is essential to enhance VNC image quality and reduce technical artifacts, such as residual CM or calcium subtractions. Moreover, the role of VNC reconstructions in the evaluation of treated arterial segments, such as stents, stentgrafts, and bypass grafts, also warrants further investigation to determine the reliability of VNC reconstructions in the post-interventional surveillance of PAD. Until such prospective diagnostic validation is established, VNC reconstructions should not be regarded as a clinically viable substitute for TNC scans.

## Figures and Tables

**Table 1:** CT scanning parameters of true non-contrast (TNC) acquisition and dual energy early arterial phase (DE-CTA) scans for virtual non-contrast (VNC) reconstructions.

Parameters	Non-Contrast Phase CT for TNC	Dual-Energy Early Arterial Phase CTA for VNC
Tube voltage (kVp)	120	A: 80, B: Sn150
Reference effective (mAs)	80	170/94
Pitch	0.6	0.7
Slice collimation (mm)	3	3
Acquisition	192 × 0.6 mm^2^	128 × 0.6 mm^2^
Rotation time (s)	0.5	0.25
Matrix	512 × 512	512 × 512
FOV	380 × 380	380 × 380
Convolution kernel	Bv40d/3	Qr36d/3
Reconstructed slice thickness (mm)	1	1
Increment (mm)	0.7	0.7

TNC, true non-contrast; VNC, virtual non-contrast; CTA, computed tomography angiography; kVp, kilovoltage peak; mAs, milliampere seconds; FOV, field of view.

**Table 2:** Structure and Likert scores for qualitative analysis of VNC images.

Overall Image Quality (4-Pont Likert Scale)
(4) excellent, equal to the quality of TNC
(3) good, little worse/almost the same quality than the quality of TNC
(2) moderate, but sufficient for diagnosis, moderately worse than the quality of TNC
(1) non-diagnostic, very poor quality compared to the quality of TNC
**Signs of Residual Contrast Medium (3-Point Likert Scale)**
For each arterial segment, separately for the right and left lower limb arteries
(3) complete removal, no signs of residual CM
(2) moderate removal, minor amount of residual CM
(1) insufficient removal, significant amount of residual CM
**Signs of Subtracted Calcifications (3-Point Likert Scale)**
For each arterial segment, separately for the right and left lower limb arteries
(3) excellent, no removal, calcified lesions are fully depicted
(2) acceptable for diagnosis, smaller areas of calcified lesions were removed
(1) insufficient for diagnosis, larger areas of calcified lesions were removed
**Assessability of Bypass Grafts (3-Point Likert Scale)**
For each arterial level, separately for the right and left lower extremity grafts
(3) excellent, the visualisation of bypass graft is of equal quality than in TNC
(2) acceptable, the visualisation of bypass graft is moderately different
(1) unsatisfactory, the visualisation of the bypass graft is highly different

CM, contrast medium.

**Table 3:** Demographic and clinical characteristics of the study population.

Demographic Data	Value
Patients (*n*)	175
Men (*n*, %)	111 (63.4%)
Women (*n*, %)	64 (36.6%)
Age (yrs)	69.3 (±9.5)
Weight (kg)	76.3 (±17.6)
Height (cm)	169.9 (±8.9)
BMI (kg/m^2^)	26.3 (±5.0)
**Anamnestic Data**	***n*** **(%)**
Previous or active smoking (*n* = 134)	109 (81.3%)
Hypertension (*n* = 171)	158 (92.4%)
Diabetes mellitus (*n* = 171)	59 (34.5%)
Hyperlipidaemia ((*n* = 171)	141 (82.5%)
Impaired renal function (*n* = 170)	55 (32.4%)
Previous stroke (*n* = 172)	25 (14.5%)
Malignant tumour (*n* = 172)	30 (17.4%)
Heart surgery or PCI (*n* = 171)	65 (38.0%)
Previous lower extremity bypass surgery (*n* = 175)	30 (17.1%)
**CTA Indication and Symptom Severity**	***n*** **(%)**
CTA Indication (elective/acute)	
Elective	153 (87.4%)
Acute	22 (12.6%)
Fontaine stages (chronic)	
I. Asymptomatic	14 (8%)
II.a Mild claudication (>200 m)	17 (9.7%)
II.b Moderate to severe claudication (<200 m)	70 (40.0%)
III. Ischemic rest pain/nocturnal leg pain	17 (9.7%)
IV. Ulcers or gangrene	35 (12.6%)
Rutherford stages (acute)	
I. Viable limb	3 (13.6%)
II.a Marginally threatened limb	10 (45.5%)
II.b Immediately threatened limb	9 (40.9%)
III. Non-salvageable limb	0

Continuous variables are described as mean ± SD, categorical variables are reported as the number (percentage). BMI, body mass index; PCI, percutaneous coronary intervention.

**Table 4:** Characteristics of the bypass grafts.

Characteristic	Overall	AFB	IFB	Crossover	FPB	PPB
Number of grafts	37	7	2	5	21	2
Bypass conduit material						
Synthetic	22	7	2	5	6	2
Autologous vein	11	0	0	0	11	0
Patency on CTA						
Patent	19	5	1	3	10	0
Occluded	18	2	1	2	11	2

AFB, aorto-femoral bypass; IFB, ilio-femoral bypass; FPB, femoro-popliteal bypass; PPB, popliteo-popliteal bypass.

**Table 5:** Comparison of attenuation (HU), image noise (SD), SNR in native arteries and in muscle and adipose tissue, and of CNR in native arteries between TNC and VNC images.

Anatomical Region	Obs.	TNC	VNC	Difference	*p*-Value
Infrarenal aorta	173				
Attenuation		50.6 ± 6.6	39.8 ± 6.3	10.8 ± 7.2	<0.0001
Image noise		19.9 ± 3.5	18.5 ± 4.2	1.5 ± 4.3	<0.0001
SNR		2.6 ± 0.6	2.3 ± 0.7	0.3 ± 0.7	<0.0001
CNR		0.04 ± 0.5	−0.5 ± 0.6	0.6 ± 0.6	<0.0001
CIA	161				
Attenuation		48.2 ± 8.6	38.4 ± 7.9	9.8 ± 8.8	<0.0001
Image noise		17.6 ± 4.5	15.76 ± 4.5	1.8 ± 5.1	<0.0001
SNR		2.9 ± 0.9	2.7 ± 1.0	0.3 ± 1.1	<0.002
CNR		−0.1 ± 0.7	−0.7 ± 0.8	0.6 ± 0.8	<0.0001
EIA	157				
Attenuation		44.9 ± 7.5	38.5 ± 7.0	6.4 ± 8.4	<0.0001
Image noise		14.7 ± 4.1	13.2 ± 4.3	1.5 ± 5.0	<0.0003
SNR		3.4 ± 2.2	3.3 ± 1.6	0.1 ± 2.3	0.53
CNR		−0.4 ± 1.0	−0.9 ± 1.0	0.5 ± 1.1	<0.0001
CFA	169				
Attenuation		45.4 ± 6.9	39.0 ± 7.5	6.3 ± 7.4	<0.0001
Image noise		14.1 ± 3.7	11.1 ± 3.5	3.0 ± 4.3	<0.0001
SNR		3.5 ± 1.4	4.2 ± 3.2	−0.7 ± 3.0	<0.004
CNR		−0.4 ± 0.8	−1.2 ± 2.9	0.8 ± 2.6	<0.0001
SFA	136				
Attenuation		42.9 ± 6.6	36.5 ± 7.6	6.4 ± 8.9	<0.0001
Image noise		10.2 ± 3.5	8.4 ± 3.6	1.7 ± 4.9	<0.0001
SNR		4.9 ± 2.7	5.3 ± 3.2	−0.4 ± 4.1	0.29
CNR		−0.9 ± 1.3	−1.9 ± 2.1	1.0 ± 2.0	<0.0001
PA	152				
Attenuation		43.3 ± 6.6	38.6 ± 5.6	4.7 ± 6.9	<0.0001
Image noise		8.8 ± 2.	6.1 ± 2.6	2.7 ± 3.2	<0.0001
SNR		5.6 ± 3.1	7.4 ± 4.0	−1.8 ± 3.4	<0.0001
CNR		−0.9 ± 1.4	−2.2 ± 2.3	1.3 ± 2.0	<0.0001
Muscle	175				
Attenuation		50.3 ± 7.0	49.5 ± 7.5	0.8 ± 6.5	0.12
Image noise		20.9 ± 4.0	15.9 ± 4.0	5.0 ± 3.4	<0.0001
SNR		2.8 ± 0.6	3.5 ± 0.9	−0.7 ± 0.7	<0.0001
Adipose tissue	175				
Attenuation		−104.3 ± 9.5	−68.2 ± 12.6	−36.1 ± 11.6	<0.0001
Image noise		18.5 ± 5.2	14.7 ± 3.5	3.8 ± 4.9	<0.0001
SNR		−5.9 ± 1.2	−4.99 ± 1.4	−1.0 ± 1.2	<0.0001

SNR, Signal-to-noise ratio; CNR, Contrast-to-noise ratio; CIA, common iliac artery; EIA, external iliac artery; CFA, common femoral artery; SFA, superficial femoral artery; PA, popliteal artery.

**Table 6:** Comparison of attenuation (HU), image noise (SD), SNR, and CNR in bypass grafts between TNC and VNC images.

Arterial Level of the Bypass Graft	Obs.	TNC	VNC	Difference	*p*-Value
Infrarenal aorta	4				
Attenuation		48.0 ± 5.8	34.3 ± 4.2	13.8 ± 9.3	0.06
Image noise		21.5 ± 3.5	17.8 ± 2.9	3.8 ± 2.2	0.04
SNR		2.3 ± 0.5	2.0 ± 0.3	0.3 ± 0.7	0.41
CNR		−0.4 ± 0.3	−1.2 ± 0.6	0.8 ± 0.7	0.12
CIA	6				
Attenuation		42.7 ± 6.3	40.5 ± 6.8	2.2 ± 8.9	0.58
Image noise		19.7± 4.8	15.8 ± 2.9	3.8 ± 5.3	0.14
SNR		2.2 ± 0.5	2.7 ± 0.1	−0.5 ± 1.2	0.41
CNR		−0.6 ± 0.5	−0.7 ± 0.7	0.1 ± 0.6	0.69
EIA	6				
Attenuation		45.5 ± 5.7	37.3 ± 5.3	8.2 ± 4.7	0.008
Image noise		16.3 ± 2.3	16.5 ± 5.4	−0.2 ± 6.0	0.95
SNR		2.9 ± 0.6	2.4 ± 0.6	0.4 ± 0.6	0.13
CNR		−0.5 ± 0.6	−1.0 ± 0.6	0.6 ± 0.6	0.05
CFA	21				
Attenuation		45.8 ± 6.7	40.4 ± 4.9	5.3 ± 4.7	<0.0001
Image noise		14.4 ± 2.9	11.8 ± 2.5	2.7 ± 3.4	0.002
SNR		3.3 ± 0.8	3.6 ± 1.0	−0.3 ± 1.1	0.20
CNR		−0.5 ± 0.7	−0.9 ± 0.8	0.5 ± 0.7	0.009
SFA	10				
Attenuation		39.5 ± 5.8	32.9 ± 4.6	6.6 ± 6.6	0.01
Image noise		11.0 ± 3.4	8.1 ± 2.9	2.9 ± 4.3	0.06
SNR		3.9 ± 1.3	4.6 ± 1.8	−0.7 ± 1.9	0.28
CNR		−1.2 ± 1.5	−2.5 ± 1.6	1.4 ± 2.3	0.09
PA	6				
Attenuation		42.8 ± 8.3	35.2 ± 3.7	7.7 ± 6.5	0.03
Image noise		10.7 ± 1.5	7.0 ± 3.4	3.7 ± 3.7	0.06
SNR		4.1 ± 1.0	6.3 ± 3.6	−2.2 ± 3.7	0.21
CNR		−0.8 ± 1.0	−3.2 ± 1.2	2.4 ± 1.4	0.008

**Table 7:** Likert score ratings for overall image quality of VNC reconstructions compared to standard TNC scans.

Overall Image Quality	Likert Scores, *n* (%)			
	Score 4	Score 3	Score 2	Score 1
overall	5 (3.5%)	139 (96.5%)	0	0
reader1	0	48 (100%)	0	0
reader2	1 (2.1%)	47 (97.9%)	0	0
reader3	4 (8.3%)	44 (91.7%)	0	0

**Table 8:** Likert score ratings for residual contrast medium (CM) and calcium subtraction of VNC reconstructions compared to standard TNC scans.

Arterial Segment	Assessment of Residual CM			Assessment of Calcium Subtraction		
	Likert Scores, *n* (%)			Likert Scores, *n* (%)		
	3	2	1	3	2	1
overall	1573 (99.3%)	11 (0.7%)	0	71 (4.5%)	1506 (95.1%)	7 (0.4%)
infrarenal aorta	143 (99.3%)	1 (0.7%)	0	10 (6.9%)	132 (91.7%)	2 (1.4%)
right CIA	143	1	0	9 (6.3%)	134 (93.1%)	1 (0.7%)
right EIA	143	1	0	7 (4.0%)	136 (94.4%)	1
right CFA	143	1	0	7	136	1
right SFA	143	1	0	4 (2.8%)	140 (97.2%)	0
right PA	143	1	0	4	136	0
left CIA	143	1	0	7	136	1
left EIA	143	1	0	7	136	1
left CFA	143	1	0	5 (3.5%)	139 (96.5%)	0
left SFA	143	1	0	5	139	0
left PA	143	1	0	6 (4.2%)	138 (95.8%)	0

**Table 9:** Interobserver agreement (Gwet’s AC2s) between the three readers in the qualitative analysis for overall image quality, removal of contrast medium and subtractions of calcified lesions.

		Gwet’s AC2	CI (95%)	*p*-Value
Overall image quality		0.990	0.98–1	<0.0001
Residual contrast medium				
	Infrarenal aorta	0.995	0.986–1	<0.0001
	right CIA	0.995	0.986–1	<0.0001
	right EIA	0.995	0.986–1	<0.0001
	right CFA	0.995	0.986–1	<0.0001
	right SFA	0.995	0.986–1	<0.0001
	right PA	0.995	0.986–1	<0.0001
	left CIA	0.995	0.986–1	<0.0001
	left EIA	0.995	0.986–1	<0.0001
	left CFA	0.995	0.986–1	<0.0001
	left SFA	0.995	0.986–1	<0.0001
	left PA	0.995	0.986–1	<0.0001
Removal of calcifications				
	Infrarenal aorta	0.957	0.925–0.988	<0.0001
	right CIA	0.958	0.925–0.988	<0.0001
	right EIA	0.969	0.943–0.996	<0.0001
	right CFA	0.964	0.936–0.992	<0.0001
	right SFA	0.981	0.961–1	<0.0001
	right PA	0.981	0.961–1	<0.0001
	left CIA	0.964	0.936–0.992	<0.0001
	left EIA	0.964	0.936–0.992	<0.0001
	left CFA	0.980	0.960–1	<0.0001
	left SFA	0.975	0.953–0.998	<0.0001
	left PA	0.975	0.952–0.998	<0.0001

AC2, second-order agreement coefficient.

**Table 10:** Radiation data of biphasic CT scans, comprising non-contrast (TNC) and dual-energy early arterial phases (DE-CTA), and single-phase DE-CTA scans.

Metric	TNC + DE-CTA (Mean ± SD)	DE-CTA Alone (Mean ± SD)	*p*-Value
DLP (mGy·cm)	989.04 ± 242.6	625.04 ± 172.6	<0.0001
CTDIvol (mGy)	7.25 ± 1.57	4.63 ± 1.46	<0.0001
ED (mSv)	5.93 ± 1.32	3.75 ± 0.96	<0.0001

DLP, dose length product; CTDIvol, CT dose index volume; ED, effective dose.

## Data Availability

The original contributions presented in this study are included in the article. Further inquiries can be directed to the corresponding author.

## Abbreviations

The following abbreviations are used in this manuscript:

PAD	Peripheral arterial disease
ABI	Ankle–brachial index
US	Ultrasound
CTA	Computed tomography angiography
MRA	Magnetic resonance angiography
DSA	Digital subtraction angiography
ALARA	As low as reasonably achievable
DE-CT	Dual-energy CT
DE-CTA	Dual-energy CTA
TNC	True non-contrast
VNC	Virtual non-contrast
CM	Contrast medium
PACS	Picture Archiving and Communicating System
kVp	Kilovoltage peak
mAs	Milliampere seconds
HU	Hounsfield unit
ROI	Region of interest
SNR	Signal-to-noise ratio
CNR	Contrast-to-noise ratio
DLP	Dose length product
CTDIvol	CT dose index volume
ED	Effective dose
SD	Standard deviation
AC2	Second-order agreement coefficient
BMI	Body mass index
PCI	Percutaneous coronary intervention
AFB	Aorto-femoral bypass
IFB	Ilio-femoral bypass
FPB	Femoro-femoral bypass
PPB	Popliteo-popliteal bypass
CIA	Common iliac artery
EIA	External iliac artery
CFA	Common femoral artery
SFA	Superficial femoral artery
PA	Popliteal artery
